# Transcranial magnetic stimulation to left VLPFC or right DLPFC promotes forgetting in working memory

**DOI:** 10.1017/S0033291725100585

**Published:** 2025-06-30

**Authors:** Mingming Qi, Huiyan Sha, Jingyan Jing, Ru Gai, Heming Gao

**Affiliations:** School of Psychology, Liaoning Normal University, Dalian, China

**Keywords:** intentional forgetting, left VLPFC, right DLPFC, rTMS, working memory

## Abstract

**Background:**

Individuals can intentionally process task-relevant information while ignoring task-irrelevant information. This study aims to investigate how to promote forgetting of task-irrelevant information through noninvasive brain stimulation, utilizing direct suppression and thought substitute inhibition mechanisms.

**Methods:**

Participants were cued to either remember task-relevant information while forgetting task-irrelevant information (IR condition) or to forget task-irrelevant items while remembering task-relevant information (IF condition). High-frequency rTMS was applied to activate the left ventrolateral prefrontal cortex (VLPFC, *n* = 32), right dorsolateral prefrontal cortex (DLPFC, *n* = 32), or vertex cortex (control condition, *n* = 32).

**Results:**

Compared to vertex stimulation, (1) The Left VLPFC stimulation promoted the memory of task-relevant information in the IR condition, and resulted in a memory deficit for the task-irrelevant information in the IF condition (active forgetting). (2) The Right DLPFC stimulation promoted the forgetting of task-irrelevant information in the IF condition (active forgetting) and facilitated the memory of task-relevant information in the IR condition.

**Conclusions:**

Stimulating left VLPFC or right DLPFC can promote active forgetting. Noninvasive brain stimulation can effectively regulate memory control.

## Introduction

Working memory (WM) is a system that temporarily stores and processes information with limited capacity (Oberauer et al., 2018; Quentin et al., 2019). Intentional forgetting the task-irrelevant information can reduce the WM load, and facilitate the processing of task-relevant information (Dames & Oberauer, 2022; Oberauer & Greve, 2022). Several studies developed working memory-directed forgetting (WM-DF) paradigms to investigate how task-relevant and task-irrelevant information are processed in WM (Sasin, Morey, & Nieuwenstein, 2017; Sasin, Sense, Nieuwenstein, & Fougnie, 2022). Participants were asked to memorize some items, and then presented with a retro-remembering/forgetting cue indicating whether these items were to-be-remembered (TBR) or to-be-forgotten (TBF). The results showed superior memory for the TBR compared to the TBF items, indicating a DF effect.

Intentional forgetting can be achieved through the direct suppression mechanism, which proposes inhibiting unwanted memory traces by halting the episodic retrieval process (Anderson & Hanslmayr, 2014; Engen & Anderson, 2018). Previous DF studies have found increased dorsolateral prefrontal cortex (DLPFC) activation in forgetting trials than in remembering trials (Rizio & Dennis, 2013; Wylie, Foxe, & Taylor, 2008). The activation of right DLPFC may suppress the hippocampal activity, resulting in inferior memory performance of TBF items (Anderson et al., 2004; Marsh et al., 2025). The thought substitution mechanism posits that the occupation of limited attentional resources by another form of memory results in the suppression of unwanted information (Engen & Anderson, 2018; Hubbard & Sahakyan, 2021, 2023). The left ventrolateral prefrontal cortex (VLPFC) is associated with the thought substitution mechanism (Benoit & Anderson, 2012), as well as memory selectivity (Badre, Poldrack, Paré-Blagoev, Insler, & Wagner, 2005). Stronger engagement of the left VLPFC can predict increased forgetting of TBF items (Benoit & Anderson, 2012).

The selective rehearsal account posits that the DF effect arises as a result of the more elaborate rehearsal of TBR items in contrast to TBF items, and the forgetting of TBF items occurs passively (Basden, Basden, & Gargano, 1993). Conversely, the inhibitory account proposes that forgetting cues initiate an active inhibition process, which prevents TBF information from being processed further and/or suppresses the memory representation of TBF information to levels below baseline (Anderson & Hanslmayr, 2014; Wylie et al., 2008). Greater activation in the prefrontal cortex was observed during TBF compared to TBR condition, suggesting frontal inhibition control induced by forgetting cues (Rizio & Dennis, 2013; Wylie et al., 2008). Furthermore, TBF items that were subsequently forgotten (TBF-F) exhibited weaker memory trace strength compared to TBR items that were subsequently forgotten (TBR-F) (Whitlock, Ding, Hubbard, & Sahakyan, 2025; Whitlock, Lo, Chiu, & Sahakyan, 2020). This finding implies that active forgetting exerts a more detrimental effect on memory than passive forgetting, thereby supporting the inhibitory account.

The forgetting cues-induced inhibitory process might merely terminate memory rehearsal, or even suppress memory representations (Levy & Anderson, 2002). Over the past decade, several studies have found that directed forgetting is difficult (Gao, Qi, & Zhang, 2019; Sasin et al., 2017; Sasin et al., 2022). Compared to the items that followed no cue or uninformative cues (where memory was assumed to passive decay), the items that followed forgetting cues showed superior memory. The forgetting cue did not promote a forgetting effect. The ironic process theory (Wegner, 1994) suggests that attempts to suppress specific thoughts may trigger an automatic monitoring process for the suppressed thought, thereby resulting in increased cognitive accessibility. The forgetting cues may initiate an inhibition process to terminate the memory rehearsal process, but not suppress the memory representation to sub-baseline level.

In this study, we modified the WM-DF task. Participants were asked to forget TBF items while remembering some TBR ones. Two memory items were simultaneously presented initially, and one of them was cued to be remembered or forgotten under either intentional remembering (IR) or intentional forgetting (IF) conditions. Specifically, following the presentation of the memory items, a selective remembering cue was presented in the IR condition, and participants were required to remember the cued-TBR item (that is intentional-TBR item) while ignoring the uncued item. The uncued item was supposed to be involuntarily forgotten and was defined as the “passive-TBF item.” In the IF condition, a selective forgetting cue was presented after the presentation of memory items. Participants were asked to forget the cued-TBF item (designated as active-TBF item) while remembering the uncued-TBR item. Notably, the TBR items might serve as substitute memories for TBF items, and were defined as substitute-TBR items. The participants might be promoted to utilize thought substitution in forgetting active-TBF items. The difference in memory performance between active-TBF items and passive-TBF items may reflect the distinct mechanisms underlying active forgetting versus passive forgetting. We hypothesized that if the inhibition process induced by selective forgetting cues can suppress the memory representation of TBF information, an enhanced active forgetting effect would be found compared to the passive forgetting effect. However, if the ironic process existed in the IF condition, the active forgetting effect would be reduced compared to the passive forgetting effect.

To investigate the differential effects of prefrontal cortex activation on memory modulation, high-frequency repetitive transcranial magnetic stimulation (rTMS) was employed to target either the left VLPFC (involved in the thought substitution mechanism) or the right DLPFC (associated with direct suppression process). We hypothesized that activating the right DLPFC using rTMS would impair the memory of TBF items by augmenting the direct suppression process, thereby providing support for inhibitory theory. Activating the left VLPFC by rTMS would enhance the memory of TBR items and impair the memory of TBF items through enhancing the thought substitution process, thus providing evidence for the role of the thought substitution mechanism in facilitating the forgetting of TBF information.

## Methods

### Participants

A total of 100 college students were recruited for this study. Participants were randomly assigned to three groups. Four participants were excluded from the formal analysis due to low accuracy (<50%) in the memory test. Finally, data from 96 participants (32 participants per group) were included in the formal analyses, specifically, the right DLPFC group (18 females, mean age = 21.31 years, *SD* = 3.19), the left VLPFC group (22 females, mean age = 20.91 years, *SD* = 2.10), and the vertex group (19 females, mean age = 20.44 years, *SD* = 2.05).

A post-hoc sensitivity analysis using MorePower (Version 6.0; Campbell & Thompson, 2012) with a sample size of 96 revealed that we had over 0.80 power to detect an effect size (*η_p_^2^*) of 0.048 for the 3 group × 4 item type analysis of variance (ANOVA) and 0.034 for the 3 group × 6 search type ANOVA. Given that the smallest effect size observed in this study was 0.094 for the 3 × 4 ANOVA and 0.052 for the 3 × 6 ANOVA, the present sample size might be adequate to guarantee sufficient statistical power for the analyses.

All participants were right-handed, had normal or corrected-to-normal vision, reported no history of neurological and psychiatric disorders or contraindications for TMS, and gave written informed consent. Participants were paid on completion of the experiment. This study was approved by the Ethics Committee of the local university and was performed in accordance with the ethical guidelines of the Declaration of Helsinki.

### Design and materials

#### The WM-DF task

**Figure 1.**
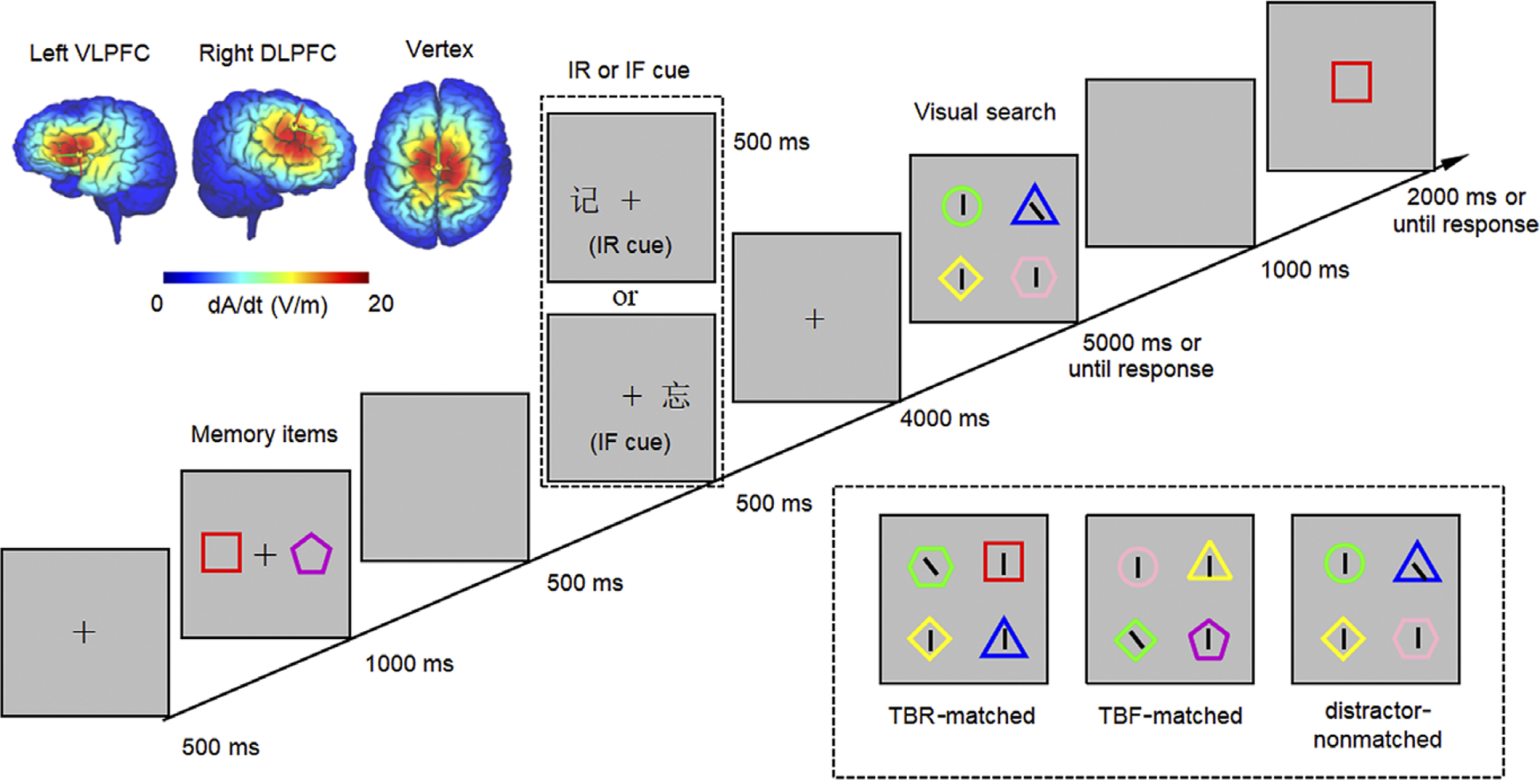
Experimental design and procedure. The transcranial magnetic stimulation was applied at the left ventrolateral prefrontal cortex (VLPFC), right dorsolateral prefrontal cortex (DLPFC), and vertex region. The simulated electric field is illustrated using SimNIBS software. The red square served as an intentional-TBR item in the IR condition or as a substitute-TBR item in the IF condition. The purple pentagon served as a passive-TBF or active-TBF item.

A modified WM-DF task was adopted. In this task, the participants were initially asked to memorize two items, one of which was subsequently cued to remember or forget in IR or IF conditions. Four types of items were obtained, i.e., intentional-TBR and passive-TBF (in IR condition), substitute-TBR and active-TBF (in IF condition) items. Then, a visual search task was followed to assess the memory trace strength associated with TBR or TBF items. Specifically, the TBR/TBF items served as distractors in the search array. Compared to the distractor-absent condition, the search RTs were longer for the distractor-presented condition (distractors matched with TBR/TBF items), demonstrating attentional capture effects (ACE) by distractors (Sasin et al., 2017; Sasin et al., 2022). A stronger memory trace of the distractor would predict a higher degree of ACE. Finally, a memory test was used to confirm whether the participants remembered the TBR items. (Figure 1).

All stimuli were presented on a gray background. The colored shapes were adopted as the memory items. The shape could be a circle, a triangle, a square, a diamond, a pentagon, or a hexagon. The color of the shapes could be red, green, blue, yellow, purple, or pink. In each trial, two colored shapes were presented on the left or right sides of the fixation. The Chinese characters “记” (meaning remember) and “忘” (meaning forget) were used as the IR cue and IF cue, respectively. In the search array of the visual search task, four colored shapes (one target and three distractors) embedded in a black line were displayed. The line embedded in the target was tilted 38° either to the left or right, while the lines in the distractors were vertical. Each item in the search array was unique in color and shape.

If an IR cue appeared at the left side of the fixation, participants were asked to remember the item on the same side (i.e., red square, intentional-TBR items) while forgetting the item on the opposite side (i.e., purple pentagon, passive-TBF items). If an IF cue appeared at the right side of the fixation, participants were asked to forget the item on the same side (i.e., blue hexagon, active-TBF items), and instead remember the item on the opposite side (i.e., green circle, substitute-TBR items) (Figure 1). Participants were told that their memory of TBR (both intentional-TBR and substitute-TBR) items would be tested in the memory test.

When the search array was presented, the participants were asked to discriminate the orientation (left or right) of the target line embedded in the objects by pressing the “Z” or “M” key on the keyboard. Two types of trials were distinguished based on the distractor match type, i.e., distractor-matched and distractor-nonmatched trials. In distractor-matched trials, one object in the search array matched both the color and shape of the memory item. In distractor-nonmatched trials, none of the objects in the search array shared any features with the memory object. Based on the combinations of cue type and distractor match type, 6 kinds of trials were obtained: intentional-TBR-matched, passive-TBF-matched, substitute-TBR-matched, active-TBF-matched, IR-nonmatched, and IF-nonmatched trials.

In the memory test, participants were instructed to indicate whether the presented item was identical to the TBR item or not by pressing “Z” or “M” on the keyboard. Only the memory of TBR (intentional-TBR and substitute-TBR) items was tested in the memory test.

#### The rTMS stimulation

rTMS is an indirect and non-invasive technique used to induce excitability changes in cortical activity (Hallett, 2000). It can change and modulate cortical activity after the stimulation period. Low-frequency stimulation (<5 Hz) has been demonstrated to suppress cortical excitability (Casula et al., 2014), whereas high-frequency stimulation (>5 Hz) enhances cortical excitability (Hallett, 2000; Jung, Shin, Jeong, & Shin, 2008). In this study, a 10 Hz rTMS was adopted to stimulate the corresponding cortex across different groups.

A TMS stimulator (Shenzhen, Yingzhi, China) with a figure-of-eight coil was utilized. The location of the coil was determined according to the international 10/20 EEG system, with F4 cited as the site for the right DLPFC, F7 cited for left VLPFC stimulation (Galli, Feurra, Pavone, Sirota, & Rossi, 2017; Rossi et al., 2001), and Cz for the vertex stimulation, which is often employed as a control condition in TMS studies due to its minimal impact on ongoing task-related processes (Jung, Bungert, Bowtell, & Jackson, 2016). The participant’s resting motor threshold (rMT) was measured before the experiment (1–2 days beforehand) and manipulated to produce the minimum TMS intensity required to produce the right abductor motor response on 5 out of 10 consecutive attempts after stimulating the left motor cortex (the C3 site) (Schutter & van Honk, 2006). The rTMS was applied at 10 Hz at 110% of the participants’ rMT. The high-frequency stimulation (10 Hz) session consisted of 33 trains, 3 s in duration, with 20 s intervals between the trains. The total number of pulses was 990 in 12.2 min. The simulated electric field is illustrated in Figure 1.

### Procedure

Upon arrival, participants received a 15-min rest. Then, the participants took the practice (32 trials) of the WM-DF task. After a short break (5 min), the participants received high-frequency rTMS for approximately 12.2 min. Thereafter, the participants performed the WM-DF task for about 45 min.

For the WM-DF task, each trial started with a 500-ms fixation. Then, two items were presented on the left and right sides of the fixation for 1000 ms. After a 500-ms blank, a cue was presented on one side of the fixation for 500 ms. After a 4000-ms blank, a search array was presented for up to 5000 ms, and disappeared once a response was submitted. After a 1000-ms blank, the memory test was presented until a response was submitted for up to 2000 ms. (Figure 1).

The formal experiment consisted of 240 trials (40 trials per condition). Participants received a short break (2 min) after each set of 60 trials. All trials were presented pseudo-randomly with the constraint that no more than three consecutive trials could be from the same cue condition.

### Statistical analysis

In the analysis of both accuracy and response time (RT, see Table 1) for the search array, only the correct trials in the memory test were included. In addition, for RT analysis, incorrect responses and reactions that were too fast or too slow (RTs that were shorter than 100 ms or longer than 2500 ms) were excluded. Finally, 8.07% of the data from the right DLPFC group, 7.29% of the data from the left VLPFC group, and 7.66% of the data from the vertex group were excluded because of deviant RTs. No differences were found for the number of trials excluded across the 3 groups [*F*(2, 93) = .114, *p* = .892]. RTs are always reported to exhibit positively skewed distributions (Baayen & Milin, 2010; Wolfe, Torralba, & Horowitz, 2002), a pattern that is also observed in visual search tasks (Wolfe et al., 2002). The RT data were subjected to a Log10 transform to correct for skewness.

**Table 1.** Mean search RTs (SD) for different search types across different groups

Group	intentional-TBR-matched	passive-TBF-matched	IR-nonmatched	substitute-TBR-matched	active-TBF-matched	IF-nonmatched
Vertex (*n* = 32)	806.3 (100.1)	783.6 (98.7)	786.8 (98.5)	842.6 (105.3)	825.2 (99.2)	788.4 (95.6)
Right DLPFC (*n* = 32)	847.3 (153.3)	802.3 (143.0)	798.1 (133.0)	851.8 (138.2)	834.4 (140.0)	820.0 (139.9)
Left VLPFC (*n* = 32)	816.5 (142.7)	784.6 (122.8)	764.8 (112.3)	843.9 (137.2)	807.3 (117.9)	790.5 (121.3)

For the visual search task, a repeated-measures ANOVA with search type (intentional-TBR-matched, passive-TBF-matched, substitute-TBR-matched, active-TBF-matched, IR-nonmatched, and IF-nonmatched) as the within-subject factor and group (right DLPFC, left VLPFC, or vertex) as the between-subject factor was performed on the search accuracy and logRT, respectively.

The ACE for different item types was calculated by subtracting the logRT of distractor-nonmatched trials from distractor-matched trials. Specifically, ACE_intentional-TBR_ = logRT_intentional-TBR-matched_ - logRT_IR-nonmatched_; ACE_passive-TBF_ = logRT_passive-TBF-matched_ - logRT_IR-nonmatched_; ACE_substitute-TBR_ = logRT_substitute-TBR-matched_ - logRT_IF-nonmatched_; ACE_active-TBF_ = logRT_active-TBF-matched_ - logRT_IF-nonmatched_. For ACE, a repeated-measures ANOVA with item type (intentional-TBR, passive-TBF, substitute-TBR, and active-TBF) as the within-subject factor and group as the between-subject factor was performed.

The memory test aimed to examine whether the participants accurately memorized the TBR items based on the cues. The memory of TBF items was not assessed in this test. Repeated-measures ANOVAs with TBR-item type (intentional-TBR, substitute-TBR) as the within-subject factor and group as the between-subject factor were performed on the accuracy.

All effects with more than one degree of freedom were adjusted for sphericity violations using the Greenhouse–Geisser correction. Fisher’s LSD correction was applied for the pairwise comparisons.

## Results

### Results of the visual search task

For the search accuracy, the ANOVA showed a main effect of trial type, *F*(5, 465) = 5.364, *p* = .001, *η_p_^2^* = .055. Pairwise comparisons showed that, (1) Accuracy was higher for IF-nonmatched compared to substitute-TBR-matched (*p* = .002) and active-TBF-matched (*p* = .047) trials. (2) Higher accuracy was found for passive-TBF-matched than IR-nonmatched trials (*p* = .032); no accuracy difference was found between intentional-TBR-matched and IR-nonmatched trials (*p* = .698). Neither the main effect of group [*F*(2, 93) = .863, *p* = .425] nor the Trial Type × Group interaction [*F*(10, 465) = 1.449, *p* = .190] was significant. The accuracies were above 98.69% (*SD* = 3.09%) across different conditions, confirming that participants completed the task seriously.

For the search logRT, a Trial Type × Group interaction was found, *F*(10, 465) = 2.535, *p* = .010, *η_p_^2^* = .052. Simple effect analyses revealed that (1) LogRTs were longer for intentional-TBR-matched compared to IR-nonmatched trials (i.e., ACE for intentional-TBR items) across the three groups (*p*s < .024). (2) Longer logRTs were observed for passive-TBF-matched than IR-nonmatched trials (i.e., ACE for passive-TBF items) in the left VLPFC group (*p* = .003), but not in the vertex and right DLPFC (*p*s > .602) groups. (3) Longer logRTs were found for both substitute-TBR-matched and active-TBF-matched trials compared to IF-nonmatched trials (i.e., ACE for both substitute-TBR and active-TBF items) across the three groups (*p*s < .019). (Mean search RTs see Table 1)

**Figure 2.**
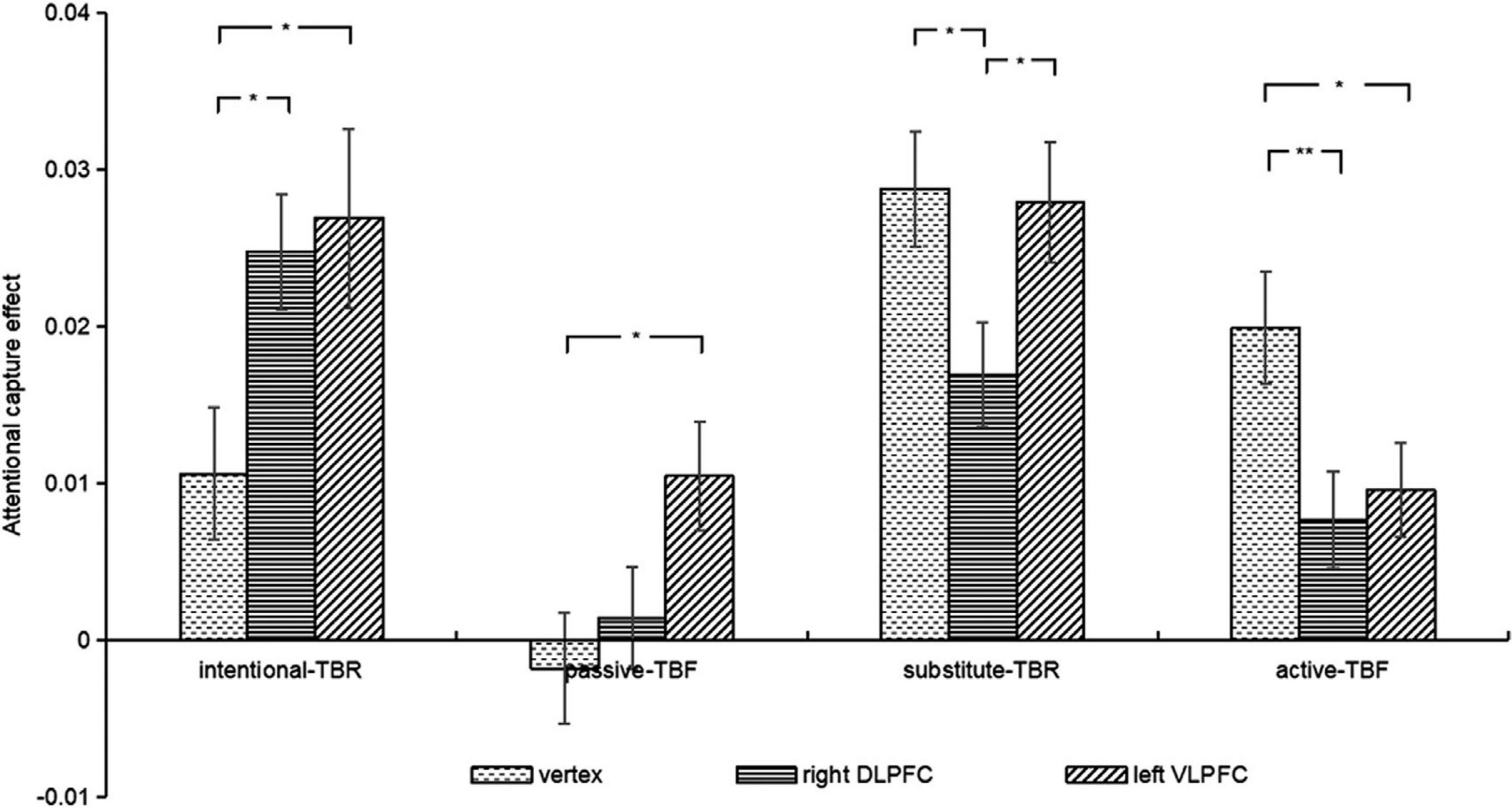
Attentional capture effect for intentional-TBR, passive-TBF, substitute-TBR, and active-TBF items across the vertex, right DLPFC, and left VLPFC groups. The error bars represented standard errors. *p < .05, ^**^p < .01.

For the ACE, an Item type × Group interaction was found, *F*(6, 279) = 4.804, *p* < .001, *η_p_^2^* = .094. Simple effect analyses revealed that: (1) Larger ACEs were found for TBR (intentional-TBR and substitute-TBR) compared to TBF (passive-TBF and active-TBF) items across the three groups (*p*s < .013); (2) ACEs were larger for substitute-TBR compared to intentional-TBR items in the vertex group (*p* = .001) but not in both right DLPFC and left VLPFC (*p*s > .129) groups; ACEs were larger for active-TBF compared to passive-TBF items in the vertex group (*p* < .001) but not in both right DLPFC and left VLPFC (*p*s > .168) groups; (3) For intentional-TBR items, ACEs were increased for both the right DLPFC (*p* = .032) and left VLPFC (*p* = .014) compared to the vertex group. No difference was found between the right DLPFC and left VLPFC groups (*p* = .745); (4) For the substitute-TBR items, larger ACEs were found for both the left VLPFC (*p* = .035) and the vertex (*p* = .023) groups compared to the right DLPFC group. No difference was found between the left VLPFC and vertex groups (*p* = .868); (5) For the passive-TBF items, larger ACEs were found for the left VLPFC compared to the vertex (*p* = .013) groups but not compared to the right DLPFC (*p* = .064) groups, no difference was found between the right DLPFC and the vertex groups (*p* = .508); (6) For the active-TBF items, decreased ACEs were found in both right DLPFC (*p* = .008) and left VLPFC (*p* = .025) groups compared to the vertex group. No difference was found between the right DLPFC and left VLPFC groups (*p* = .681) (Figure 2).

### Results of the memory test

The accuracies of the memory test were above 93.45% (*SD* = 5.19%) across different conditions. The ANOVA showed that neither the main effects nor the item type × Group interaction was significant, *F*s < 1.575, *p*s > .212. These results indicated that there were no significant differences in the accuracy of the memory test across different conditions and among the three groups.

## Discussion

Search RTs were longer for distractor-matched trials compared to distractor-nonmatched trials (i.e., ACE) for all item types except for passive-TBF items in the right DLPFC and vertex groups. These memory items functioning as distractors in the search array captured participants’ attention, indicating that these items were kept in WM. Importantly, TBR items exhibited increased ACEs compared to TBF items across all groups, suggesting that TBR items exhibited superior memory trace in comparison to TBF items (i.e., DF effect). These findings indicate that the participants successfully manipulated information stored in WM according to the cues.

ACEs for intentional-TBR items in IR conditions were increased in both the left VLPFC and right DLPFC groups compared to the vertex group, suggesting that the activation of right DLPFC and left VLPFC exerted a positive effect on selectively memorizing intentional-TBR items. The right DLPFC has been associated with active memory suppression (Anderson & Hanslmayr, 2014; Benoit & Anderson, 2012). Intentional forgetting might free WM capacity and facilitate memory performance for the remaining TBR items in WM (Dames & Oberauer, 2022; Lewis-Peacock, Kessler, & Oberauer, 2018). The right DLPFC biased processing towards relevant information compared to irrelevant information (Feredoes, Heinen, Weiskopf, Ruff, & Driver, 2011; Jackson, Feredoes, Rich, Lindner, & Woolgar, 2021). Consistent with these studies, the right DLPFC stimulation might promote the memory suppression of TBF items, thereby freeing WM capacity, and leading to superior memory trace of intentional-TBR items compared to the vertex stimulation. The left VLPFC has been associated with memory encoding selectivity (Badre et al., 2005) or thought substitution (Benoit & Anderson, 2012; Engen & Anderson, 2018), and activating this region promotes memory formation (Medvedeva et al., 2021; Weintraub-Brevda & Chua, 2019). The greater ACE of intentional-TBR items observed in the left VLPFC group compared to the vertex group might be attributed to improved selective rehearsal facilitated by activation of the left VLPFC.

For the substitute-TBR items in IF conditions, both the vertex group and left VLPFC group exhibited larger ACEs compared to the right DLPFC group. In the IF condition, participants were instructed to forget the active-TBF items while remembering the substitute-TBR items. This selective recollection of substitute-TBR items could be considered as the thought substitution process for active-TBF items. Notably, in the vertex group, greater ACE was found for substitute-TBR items compared to the intentional-TBR items, indicating memory facilitation attributed to this selective rehearsal process. Furthermore, the left VLPFC stimulation did not promote the memory trace of substitute-TBR items compared to the vertex stimulation. These findings imply that selective rehearsal exerts a substantial influence on memory formation, whereas the left VLPFC stimulation does not yield any additional memory enhancement.

The right DLPFC has been associated with the direct suppression of task-irrelevant information, thereby saving cognitive resources for processing relevant information (Jackson et al., 2021; Oehrn et al., 2018). We expected that the right DLPFC stimulation would promote the memory of substitute-TBR items (reflected by larger ACE) compared to the vertex stimulation. However, contrary to our expectations, the right DLPFC stimulation did not yield any additional effects on the memory formation for substitute-TBR items and even resulted in inferior memory compared to the vertex stimulation. The activation of right DLPFC was found to be associated with a reduction in hippocampal activity (Anderson & Hulbert, 2021; Benoit & Anderson, 2012), indicating memory inhibition for the task-irrelevant information. It is plausible that stimulating the right DLPFC may potentially suppress the hippocampal activity and negatively impact the selective rehearsal process of the substitute-TBR items. Consequently, compared to the vertex group, the right DLPFC group exhibited inferior memory trace of substitute-TBR items.

For the passive-TBF items in the IR condition, ACE was absent in both the vertex group and the right DLPFC group. Sasin et al. (2017) found that TBF items captured attention in the absence of any TBR items, thereby suggesting that TBF items were not completely forgotten. In this study, the intentional-TBR item was memorized while the passive-TBF item was forgotten in the IR condition. The selective rehearsal of intentional-TBR items may potentially lead to memory impairment of the passive-TBF items through the thought substitution inhibition. Importantly, the absence of ACE for the passive-TBF items suggests their removal from working memory.

However, the left VLPFC group exhibited an ACE for passive-TBF items, suggesting that these items were not completely forgotten. Additionally, a larger ACE was found for the left VLPFC group compared to the vertex group. These results suggest that the left VLPFC stimulation did not promote the forgetting effect of passive-TBF items compared to the vertex group; instead, it resulted in a memory enhancement for these items. The activation of left VLPFC facilitated cognitive resource allocation towards intentional-TBR items, without exerting memory impairment on passive-TBF items. Individuals may involuntarily process task-irrelevant information when attentional resources are sufficient (Lavie, Hirst, de Fockert, & Viding, 2004). Similarly, in this study, we speculated that the intentional-TBR items might be adequately processed and selectively rehearsed with less intensity. Consequently, attentional resources recruited by left VLPFC activation may have inadvertently been allocated to passive-TBF items, resulting in enhanced memory of passive-TBF items in the left VLPFC group.

For the active-TBF items, ACE was found across all groups, suggesting these items were not completely forgotten. According to the inhibitory account, forgetting cues could trigger an active inhibition process to terminate the memory rehearsal or suppress the memory representation to sub-baseline level. Notably, active-TBF items showed superior memory compared to the passive-TBF items in the vertex group, suggesting that passive forgetting was more effective than active forgetting. In accordance with the ironic process theory, forgetting cues do not facilitate a greater degree of forgetting compared to the passive decay of memory traces. Rather, they may trigger a re-alerting process that enhances the salience of TBF items, thereby increasing their memory accessibility to a certain extent (Gao et al., 2019; Schindler & Kissler, 2018). In this study, the IF cue might have re-alerted the active-TBF items, resulting in a memory ironic effect. In addition, although the memory of passive-TBF items in the IR condition was supposed to be passive decay, in fact, it might be suppressed through thought substitute inhibition. The combined effects of thought substitute inhibition in the IR condition and cue-related ironic monitoring effect in the IF condition resulted in a more pronounced forgetting effect for the passive-TBF compared to active-TBF items.

ACE for the active-TBF items was decreased for both the right DLPFC and left VLPFC groups compared to the vertex group, indicating that the activation of the right DLPFC and left VLPFC promoted the forgetting of the active-TBF items. The right DLPFC is involved in the direct suppression process of intentional forgetting, leading to the forgetting of suppressed information (Benoit & Anderson, 2012; Gamboa, von Wegner, Behrens, & Steinmetz, 2018). Consistent with these findings, the present results suggest that the right DLPFC activation facilitates memory inhibition, thereby amplifying the forgetting effect for active-TBF items. The left VLPFC plays an important role in suppressing unwanted information through thought substitution (Benoit & Anderson, 2012). In this study, activating left VLPFC might facilitate memory impairment of the active-TBF items by boosting the thought substitution process. These results suggest that the memory of active-TBF items is not merely subject to passive decay, instead, it can be modulated by TMS intervention, thereby providing support for the inhibitory account. Notably, as there was no baseline condition to measure the passive decay of memory items, it was unclear whether the memory of active-TBF items was suppressed to sub-baseline. Although TMS intervention promoted forgetting of active-TBF items, the presence of ACE for these items indicates that they were not entirely forgotten.

In sum, the activation of right DLPFC might lead to the improvement of direct suppression of active-TBF items, thereby facilitating the selective rehearsal of TBR items in conditions involving explicit remember cues. Activation of left VLPFC could improve the thought substitution process, leading to enhanced memory formation for TBR items and memory inhibition for TBF items in conditions involving explicit forgetting cues. Forgetting is an active process that can be modulated by noninvasive brain stimulation.

## Data Availability

The datasets generated during the current study are available in the OSF repository, https://osf.io/7bnt5/?view_only=8e275904e013438a9d98a65852b6e249.

## References

[r1] Anderson, M. C., & Hanslmayr, S. (2014). Neural mechanisms of motivated forgetting. Trends in Cognitive Sciences, 18(6), 279–292. 10.1016/j.tics.2014.03.002. 24747000 PMC4045208

[r2] Anderson, M. C., & Hulbert, J. C. (2021). Active forgetting: Adaptation of memory by prefrontal control. Annual Review of Psychology, 72, 1–36. 10.1146/annurev-psych-072720-094140. 32928060

[r3] Anderson, M. C., Ochsner, K. N., Kuhl, B., Cooper, J., Robertson, E., Gabrieli, S. W., & Gabrieli, J. D. (2004). Neural systems underlying the suppression of unwanted memories. Science, 303(5655), 232–235. 10.1126/science.1089504. 14716015

[r4] Baayen, R. H., & Milin, P. (2010). Analyzing reaction times. International Journal of Psychological Research, 3(2), 12–28. 10.21500/20112084.807.

[r5] Badre, D., Poldrack, R. A., Paré-Blagoev, E. J., Insler, R. Z., & Wagner, A. D. (2005). Dissociable controlled retrieval and generalized selection mechanisms in ventrolateral prefrontal cortex. Neuron, 47, 907–918. 10.1016/j.neuron.2005.07.023. 16157284

[r6] Basden, B. H., Basden, D. R., & Gargano, G. J. (1993). Directed forgetting in implicit and explicit memory tests: A comparison of methods. Journal of Experimental Psychology: Learning, Memory, and Cognition, 19(3), 603–616. 10.1037/0278-7393.19.3.603.

[r7] Benoit, R. G., & Anderson, M. C. (2012). Opposing mechanisms support the voluntary forgetting of unwanted memories. Neuron, 76(2), 450–460. 10.1016/j.neuron.2012.07.025. 23083745 PMC3480638

[r8] Campbell, J. I., & Thompson, V. A. (2012). MorePower 6.0 for ANOVA with relational confidence intervals and Bayesian analysis. Behavior Research Methods, 44(4), 1255–1265. 10.3758/s13428-012-0186-0. 22437511

[r9] Casula, E. P., Tarantino, V., Basso, D., Arcara, G., Marino, G., Toffolo, G. M., & Bisiacchi, P. S. (2014). Low-frequency rTMS inhibitory effects in the primary motor cortex: Insights from TMS-evoked potentials. NeuroImage, 98, 225–232. 10.1016/j.neuroimage.2014.04.065. 24793831

[r10] Dames, H., & Oberauer, K. (2022). Directed forgetting in working memory. Journal of Experimental Psychology. General, 151(12), 2990–3008. 10.1037/xge0001256. 35696174

[r11] Engen, H. G., & Anderson, M. C. (2018). Memory control: A fundamental mechanism of emotion regulation. Trends in Cognitive Sciences, 22(11), 982–995. 10.1016/j.tics.2018.07.015. 30122359 PMC6198111

[r12] Feredoes, E., Heinen, K., Weiskopf, N., Ruff, C., & Driver, J. (2011). Causal evidence for frontal involvement in memory target maintenance by posterior brain areas during distracter interference of visual working memory. Proceedings of the National Academy of Sciences, 108(42), 17510–17515. 10.1073/pnas.1106439108. PMC3198359 21987824

[r13] Galli, G., Feurra, M., Pavone, E. F., Sirota, M., & Rossi, S. (2017). Dynamic changes in prefrontal cortex involvement during verbal episodic memory formation. Biological Psychology, 125, 36–44. 10.1016/j.biopsycho.2017.02.008. 28238886

[r14] Gamboa, O. L., von Wegner, F., Behrens, M., & Steinmetz, H. (2018). The challenge of forgetting: Neurobiological mechanisms of auditory directed forgetting. Human Brain Mapping, 39, 249–263. 10.1002/hbm.23840. 29080232 PMC6866323

[r15] Gao, H., Qi, M., & Zhang, Q. (2019). Forgetting cues are ineffective in promoting forgetting in the item-method directed forgetting paradigm. International Journal of Psychophysiology, 144, 25–33. 10.1016/j.ijpsycho.2019.07.004. 31377379

[r16] Hallett, M. (2000). Transcranial magnetic stimulation and the human brain. Nature, 406(6792), 147–150. 10.1038/35018000. 10910346

[r17] Hubbard, R. J., & Sahakyan, L. (2021). Separable neural mechanisms support intentional forgetting and thought substitution. Cortex, 142, 317–331. 10.1016/j.cortex.2021.06.013. 34343901

[r18] Hubbard, R. J., & Sahakyan, L. (2023). Differential recruitment of inhibitory control processes by directed forgetting and thought substitution. The Journal of Neuroscience, 43(11), 1963–1975. 10.1523/JNEUROSCI.0696-22.2023. 36810228 PMC10027038

[r19] Jackson, J. B., Feredoes, E., Rich, A. N., Lindner, M., & Woolgar, A. (2021). Concurrent neuroimaging and neurostimulation reveals a causal role for dlPFC in coding of task-relevant information. Communications Biology, 4(1), 588. 10.1038/s42003-021-02109-x. 34002006 PMC8128861

[r20] Jung, J., Bungert, A., Bowtell, R., & Jackson, S. R. (2016). Vertex stimulation as a control site for Transcranial magnetic stimulation: A concurrent TMS/fMRI study. Brain Stimulation, 9(1), 58–64. 10.1016/j.brs.2015.09.008. 26508284 PMC4720218

[r21] Jung, S. H., Shin, J. E., Jeong, Y. S., & Shin, H. I. (2008). Changes in motor cortical excitability induced by high-frequency repetitive transcranial magnetic stimulation of different stimulation durations. Clinical Neurophysiology, 119(1), 71–79. 10.1016/j.clinph.2007.09.124. 18039593

[r22] Lavie, N., Hirst, A., de Fockert, J. W., & Viding, E. (2004). Load theory of selective attention and cognitive control. Journal of Experimental Psychology: General, 133(3), 339–354. 10.1037/0096-3445.133.3.339. 15355143

[r23] Lewis-Peacock, J. A., Kessler, Y., & Oberauer, K. (2018). The removal of information from working memory. Annals of the New York Academy of Sciences, 1424(1), 33–44. 10.1111/nyas.13714. 29741212

[r24] Levy, B. J., & Anderson, M. C. (2002). Inhibitory processes and the control of memory retrieval. Trends in Cognitive Sciences, 6(7), 299–305. 10.1016/s1364-6613(02)01923-x. 12110363

[r25] Marsh, L. C., Apšvalka, D., Kikuchi, H., Abe, N., Kawaguchi, J., Kopelman, M. D., & Anderson, M. C. (2025). Prefrontally mediated inhibition of memory systems in dissociative amnesia. Psychological Medicine, 54(16), 1–9. 10.1017/S0033291724003040. PMC11779556 39773554

[r26] Medvedeva, A., Saw, R., Silvestri, C., Sirota, M., Fuggetta, G., & Galli, G. (2021). Offset-related brain activity in the left ventrolateral prefrontal cortex promotes long-term memory formation of verbal events. Brain Stimulation, 14(3), 564–570. 10.1016/j.brs.2021.03.002. 33722660

[r27] Oberauer, K., & Greve, W. (2022). Intentional remembering and intentional forgetting in working and long-term memory. Journal of Experimental Psychology: General, 151(3), 513–541. 10.1037/xge0001106. 34570560

[r28] Oberauer, K., Lewandowsky, S., Awh, E., Brown, G. D. A., Conway, A., Cowan, N., & Ward, G. (2018). Benchmarks for models of short-term and working memory. Psychological Bulletin, 144(9), 885–958. 10.1037/bul0000153. 30148379

[r29] Oehrn, C. R., Fell, J., Baumann, C., Rosburg, T., Ludowig, E., Kessler, H., & Axmacher, N. (2018). Direct electrophysiological evidence for prefrontal control of hippocampal processing during voluntary forgetting. Current Biology, 28, 3016–3022. 10.1016/j.cub.2018.07.042. 30197086

[r30] Quentin, R., King, J. R., Sallard, E., Fishman, N., Thompson, R., Buch, E. R., & Cohen, L. G. (2019). Differential brain mechanisms of selection and maintenance of information during working memory. Journal of Neuroscience, 39(19), 3728–3740. 10.1523/JNEUROSCI.2764-18.2019. 30833510 PMC6510345

[r31] Rizio, A. A., & Dennis, N. A. (2013). The neural correlates of cognitive control: Successful remembering and intentional forgetting. Journal of Cognitive Neuroscience, 25(2), 297–312. 10.1162/jocn_a_00310. 23066730

[r32] Rossi, S., Cappa, S. F., Babiloni, C., Pasqualetti, P., Miniussi, C., Carducci, F., & Rossini, P. M. (2001). Prefrontal cortex in long-term memory: An “interference” approach using magnetic stimulation. Nature Neuroscience, 4(9), 948–952. 10.1038/nn0901-948. 11528428

[r33] Sasin, E., Morey, C. C., & Nieuwenstein, M. (2017). Forget me if you can: Attentional capture by to-be-remembered and to-be-forgotten visual stimuli. Psychonomic Bulletin & Review, 24(5), 1643–1650. 10.3758/s13423-016-1225-0. 28074450 PMC5643359

[r34] Sasin, E., Sense, F., Nieuwenstein, M., & Fougnie, D. (2022). Training modulates memory-driven capture. Attention, Perception & Psychophysics, 84(5), 1509–1518. 10.3758/s13414-022-02508-0. PMC9232407 35680783

[r35] Schindler, S., & Kissler, J. (2018). Too hard to forget? ERPs to remember, forget, and uninformative cues in the encoding phase of item-method directed forgetting. Psychophysiology, 55, e13207. 10.1111/psyp.13207. 30112763

[r36] Schutter, D. J., & van Honk, J. (2006). An electrophysiological link between the cerebellum, cognition and emotion: Frontal theta EEG activity to single-pulse cerebellar TMS. NeuroImage, 33(4), 1227–1231. 10.1016/j.neuroimage.2006.06.055. 17023183

[r37] Wegner, D. M. (1994). Ironic processes of mental control. Psychological Review, 101(1), 34–52. 10.1037/0033-295x.101.1.34. 8121959

[r38] Weintraub-Brevda, R. R., & Chua, E. F. (2019). Transcranial direct current stimulation over the right and left VLPFC leads to differential effects on working and episodic memory. Brain and Cognition, 132, 98–107. 10.1016/j.bandc.2019.03.005. 30939358

[r39] Whitlock, J., Ding, H., Hubbard, R., & Sahakyan, L. (2025). Delayed testing in directed forgetting dissociates active and passive forms of forgetting. Journal of Experimental Psychology. Learning, Memory, and Cognition, 51(5), 774–790. 10.1037/xlm0001394 39418448

[r40] Whitlock, J., Lo, Y. P., Chiu, Y. C., & Sahakyan, L. (2020). Eye movement analyses of strong and weak memories and goal-driven forgetting. Cognition, 204, 104391. 10.1016/j.cognition.2020.104391. 32717426

[r41] Wolfe, J. M., Torralba, A., & Horowitz, T. S. (2002). Remodeling visual search: How gamma distributions can bring those boring old RTs to life. Journal of Vision, 2(7), 735. 10.1167/2.7.735.

[r42] Wylie, G. R., Foxe, J. J., & Taylor, T. L. (2008). Forgetting as an active process: An FMRI investigation of item-method-directed forgetting. Cerebral Cortex, 18(3), 670–682. 10.1093/cercor/bhm101. 17617657

